# 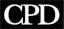 How Emergency Nurses Develop Resilience in the Context of Workplace Violence: A Grounded Theory Study

**DOI:** 10.1111/jnu.12668

**Published:** 2021-05-06

**Authors:** Chin‐Yen Han, Li‐Chin Chen, Chun‐Chih Lin, Suzanne Goopy, Hui‐Ling Lee

**Affiliations:** ^1^ Associate Professor Department of Nursing, and Clinical Competency Center Chang Gung University of Science and Technology; Associate Research Fellow (joint appointment) Department of Nursing, Chang Gung Memorial Hospital at Linkou Taiwan Republic of China; ^2^ Director Department of Nursing New Taipei Municipal Tuncheng Hospital Chang Gung Memorial Hospital; Assistant Professor, Department of Nursing, Chang Gung University of Science and Technology Taiwan Republic of China; ^3^ Associate Professor Department of Nursing Chang Gung University of Science and Technology; Associate Research Fellow (joint appointment) Department of Nursing, Chang Gung Memorial Hospital at Linkou Taiwan Republic of China; ^4^ Senior Teaching Fellow and Programme Coordinator Usher institute University of Edinburgh, UK, and Adjunct Associate Professor at University of Calgary Canada; ^5^ Lecturer Department of Nursing Chang Gung University of Science and Technology Taiwan Republic of China

**Keywords:** Emergency nursing, grounded theory, nursing commitment, resilience, workplace violence

## Abstract

**Purpose:**

To understand how emergency nurses develop resilience in the context of workplace violence.

**Design:**

This study employed grounded theory methodology. Thirty nurses from three hospital emergency departments in Taiwan were interviewed between August and December 2018.

**Methods:**

Semistructured interviews were used to collect data. Interviews were audio‐recorded and transcribed verbatim.

**Findings:**

The process through which emergency nurses who had experienced workplace violence developed resilience took place in three stages: the release of emotions after the assault; the interpretation of conflicting thoughts and actions; and the establishment of strategies to cope with workplace violence in the future. The core theme was the motivating role of professional commitment to emergency patient care.

**Conclusions:**

The results of this study can inform the development of support systems to enhance the resilience of nurses experiencing workplace violence by alerting healthcare administrators and governing institutions to their needs.

**Clinical Relevance:**

Emergency nurses viewed professional growth and professional commitment as an invisible motivator in the development of resilience following an encounter with workplace violence.

International research demonstrates the adverse impact of workplace violence (WPV) on nurses (Copeland & Henry, 2017; Hamdan & Hamra, 2017; Jeong & Kim, 2018; Lee et al., 2020; Li, Chao, M., & Shih, 2018; Wright‐Brown, Sekula, Gillespie, & Zoucha, 2016). Apart from the immediate physical and psychological effects on individuals (Han et al., 2017; Hsieh, Hung, Wang, Ma, & Chang, 2016), WPV can have wider social and economic consequences. In 2016, WPV is estimated to have cost U.S. hospitals and health systems approximately $2.7 billion (American Hospital Association, 2017). Nurses are at high risk for WPV (Ferri, Silvestri, Artoni, & Lorenzo, 2016; Shi et al., 2017), and those working in emergency departments (EDs) experience the highest rate (Emergency Nurses Association, 2014; Shafran‐Tikva, Zelker, Stern, & Chinitz, 2017; Shi et al., 2017).

## Background

WPV is a persistent problem across health care and especially nursing. The World Health Organization has defined WPV as incidents in which staff are abused, threatened, or assaulted in circumstances related to their work (World Health Organization, 2002). The International Council of Nurses Position Statement on WPV draws our attention to an even more nuanced definition that indicates the range of risks that nurses in particular face: “Incidents where staff are abused, threatened or assaulted in circumstances related to their work, including commuting to and from work, involving an explicit or implicit challenge to their safety, well‐being or health” (International Council of Nurses, 2017). ED nurses encounter incidents of WPV on a daily or weekly basis (Copeland & Henry, 2017; Li et al., 2018) and are at 1.7 times higher risk for injury compared with other professions (Groenewold et al., 2018). Two thirds of ED nurses are worried that assaults may occur at any time, while 38% to 45% of nurses experience moderate to severe depression after an incident of WPV (Gong et al., 2014). This can lead to a loss of passion and satisfaction with work (Copeland & Henry, 2017; Han et al., 2017; Li et al., 2017), a decision to leave nursing as a profession (Li et al., 2017; Partridge & Affleck, 2017), and deterioration in the quality of care and services delivered to patients (Hamdan & Hamra, 2017; Han et al., 2017; Wright‐Brown et al., 2016).

Resilience has been identified as one of the most important factors in successfully adapting to and recovering from exposure to adversity. Some individuals can proactively develop and enact strategies to become resilient and adapt to stressful environments (Frankenberg, Sikoki, Sumantri, Suriastini, & Thomas, 2013). The ED is one such environment, where WPV against nurses is common. ED nurses interpret WPV as an inevitable part of their work as long as they remain in the profession (Han et al., 2017; Li et al., 2018). In a recent study in Taiwan, 92.9% of 407 ED nurses reported experiencing WPV within the previous 2 years (Lee et al., 2020). This figure is consistent with findings from studies in Australia with 88.1% (Partridge & Affleck, 2017), Canada with 86.5% (Lemelin et al., 2009), Palestine with 76% (Hamdan & Hamra, 2015), Jordan with 75% (ALBashtawy & Aljezawi, 2016), the United States with 70% (Gacki‐Smith et al., 2009), and Turkey with 75% (Pinar & Ucmak, 2010). However, little research attention has been paid to how ED nurses develop resilience after experiencing WPV.

## Methods

### Grounded Theory Methodology

The aim of the current study was to investigate the process through which nurses develop resilience in the aftermath of WPV. The study used grounded theory methodology to generate insights into the nurses’ views and interpretations of WPV. Grounded theory is generally employed to investigate connections that have yet to be clearly defined or research fields and topics that are little developed (Corbin & Strauss, 2014; Strauss & Corbin, 1994). Grounded theory involves gaining meaningful insights from comprehensively collected data and forming a theory from this information. It is based on symbolic interactionism (Glaser, 1998; Glaser & Strauss, 1967), a theory derived from a social constructionist perspective that focuses on the interaction between individuals and society. Grounded theory aims to produce a summative account of the phenomenon of interest by systematically collecting and analyzing data in order to develop a practical or a conceptual theory (Glaser & Strauss, 1967; Strauss & Corbin, 1994). The processes of data collection, analysis, and theory construction are interconnected and evolve mutually during the research procedure.

### Participants

The study was conducted across three emergency departments in Taiwan. Eligibility criteria for participation were (a) registered nurses providing care for emergency patients, (b) had experienced WPV in the ED, (c) were 20 years of age and older, and (d) were willing to participate in the study.

### Data Collection

Thirty participants who met the eligibility criteria participated in semistructured interviews. Individual in‐depth face‐to‐face semistructured interviews were undertaken by the one researcher for the entire study. All interviews started with the same question: “What is your experience with WPV in the ED?” From this point, the interviews continued following a semistructured format whereby questions, guided by existing constructs, were asked in ways that were responsive to the participants’ experiences as expressed by their responses to the questions posed. In this way, beyond the first formally structured question, questions were semistructured, allowing participants to effectively lead the direction of the interviews under the broad guidance of the interviewer. In this way interviews were conducted based on the principles of reciprocity and reflexivity as they reflected the experiences of the participants through their responses as well as the interviewers’ understanding of the concept of WPV resilience taken from the analysis of previously completed interviews. In keeping with the emergent and inductive nature of the grounded theory methodology used in this study, participants were recruited initially through purposive sampling and subsequently through both purposive and theoretical sampling, where the selection of potential participants was guided by the emerging concepts and theory (Glaser, 1998; Glaser & Strauss, 1967). Purposive sampling provides the initial data that the researcher analyzes. Theoretical sampling is used to identify and follow clues from the analysis, fill gaps, clarify uncertainties, check hunches, and test interpretations as the study progresses (Corbin & Strauss, 2014). In the present study, the theoretical sampling was done by analyzing the original transcripts. The researchers collected data until saturation was achieved. Interviews were audio‐recorded and ranged in length from 47 to 74 min. Data were collected between August and December 2018.

### Data Analysis

As noted above, the very nature of grounded theory methodology makes it difficult to divide the research neatly into distinct categories of data collection and analysis. For heuristic purposes, this section presents a brief reflexive account of the analytic process, which was guided by theoretical coding. The relationship between the statement made (the interview quote) and the code given, and the derivation from this to the final categories, are shown (Corbin & Strauss, 2014). Patterns were sought as codes were allocated, and concepts emerged as part of the conversation between coding and analysis. As the concepts emerged, they guided our theoretical sampling of new data and further elaboration of the concepts (Corbin & Strauss, 2014; Strauss & Corbin, 1994).

### Ethical Approval

The study was approved by the Institutional Review Board of the hospitals from which participants were recruited. All participants were informed about the study, were provided with written information, and gave written consent to participate. Participation was voluntary, anonymous, and did not influence subsequent employment. Participants were able to withdraw at any time during the research without penalty. No identifying information was recorded.

### Rigor

Grounded theory provides an exceptionally strong basis for ensuring rigor (creditability, auditability, and fittingness; Corbin & Strauss, 2014; Strauss & Corbin, 1994). Because researchers implement grounded theory methodology in different ways (Cutcliffe, 2000), the procedures employed in the present study have been explained to demonstrate how rigor was maintained. In the present study, two sets of researchers independently coded responses for thematic content analysis, and any disagreements were discussed until consensus was obtained. The decision‐making process and the rationale for decisions made were contemporaneously documented by researchers throughout the study, since knowing and understanding what and how decisions are made as part of the research process are important to ensure rigor in any grounded theory study (Corbin & Strauss, 2014).

## Results

A total of 30 emergency nurses, 24 females and 6 males, were interviewed in the study. The participants’ work experience in the nursing profession ranged from 6 months to 28 years. Thematic analysis of the data identified three stages in the trajectory of these nurses’ recovery from the experience of WPV: (a) the release of emotions after the assault; (b) the interpretation of conflicting thoughts and actions; and (c) the establishment of strategies to cope with WPV events. Each of these is elaborated below.

### Stage 1: The Release of Emotions After the Assault

The emotional and physio‐psychological responses of emergency nurses after encountering WPV included crying, anger, and nightmares, which affected their personal and professional lives. The emotional aftermath of the event could be projected onto the patients for whom they were caring, could be reflected inwardly to themselves, or could affect their feelings about their work and devotion to their profession. For example, nurses described treating their patients in the way they had been treated during the incident of WPV, although their accounts revealed they were aware of the inappropriateness of these actions. As one nurse indicated:

When the assault first happened, for a period of time I couldn’t help but cry (when I think back about being rudely spoken to), and was rude to patients when on my rounds. I can’t justify my own actions, but I just couldn’t control my offensiveness toward the patients. I used to be a nice ED nurse. (N5)

Nurses inwardly reflected on their emotional responses after the event and tried to find an explanation for it. They questioned their professional abilities in the hope of finding some sort of emotional closure. For example:

This occurred when I was a new nurse. The patient’s family was aggressive in blaming me. I didn’t know what to do, and couldn’t bring myself to tell anyone about it. I cried continually and was angry when I got back to my dorm, and questioned myself about whether ED was my first choice of work in nursing. I am passionate about working in the ED. (N12)

Participants described their emotional response at the time of the WPV, and pondered the disconnection between their commitment and the occurrence of the event. Apart from experiencing negative moods and physio‐psychological reactions, they found it difficult to accept that the verbal and/or physical violence had occurred despite their dedication and the effort they put into caring for patients. They were also worried about the negative impact these emotions could have on their care for other patients in the ED, as illustrated in the following account:

I was hit in the back by a female patient who tore my glasses off and broke (them). I was furious. . . . In the ED, when we type the note we are usually facing away from the patients, and I got really suspicious whenever someone was behind me, thinking that the person might hit me. I got complaints from the next shift saying that I kept forgetting things. I was so afraid that I might make terrible mistakes and put the other patients in danger. (N2)

### Stage 2: Interpretation of Conflicting Thoughts and Actions

The emergency nurses reported that it was difficult for them to decide how to respond in the aftermath of an incident of WPV, especially after careful consideration of the potential consequences of their decisions and actions. During this stage, the nurses seem to have experienced a conflict between the practical and the ideal, and arguably chose to follow the path of least resistance. The decisions made after this careful reflection might not have been optimal, but reflected the individuals’ interpretation of their best interests. For example:

I was so angry and wanted to sue the family, but was also terrified of what he might do. I was often on the evening shift and it was easy for me to get jumped by him. Even if I wanted to pursue this through [the] court, it would be long and tiresome and I only had myself to turn to. In the end, I just had to tell myself to let it go. (N11)

Once I had a drunken patient who assaulted me. I was so mad and decided to sue. But when I sat in the police station to have the statements taken, I got so scared and was shivering all over when I saw the cuffed‐up patient giving me that look. I just wished that I would never see him again in my life, let alone [sue] him. (N1)

Thus, the participants adopted a passive approach to dealing with the aftermath of these violent events in order to achieve an emotional adjustment to the overall environment. During interviews, emergency nurses reported feeling powerless at work but found a way to justify continuing to work in the ED by “normalizing” the experience. The following accounts were typical.

After some time I just learned to put things behind, and maybe see it as the usual daily stuff. I was lucky that the patient who assaulted me was mentally normal. A while ago my colleague was assaulted by a psychotic patient, and wasn’t it just his luck! Well, you just have to learn to ignore it and not to care too much to hang in this job. (N10)

Well, . . . every job gets to deal with bad customers. At the end of the day, I just have to stick to my morals at work and give the patients my best care. This is what motivates me to keep working in the ED unit all these years. (N17)

### Stage 3: The Establishment of Strategies to Cope With WPV Events

Work experience and growth along the career path were crucial to emergency nurses’ ability to reconcile the experience of WPV with their professional responsibility of care in the unique work environment of the ED. Participants reported that, as they gained more experience at work, they adopted a new perspective on WPV. As is demonstrated in the following extracts, they drew on their experience and the communication skills they had developed during patient care to identify potential triggers and perpetrators of WPV as well as appropriate management techniques in the event of an incident. Especially when nurses realized that they encountered acts of violence less frequently, they felt that they had grown professionally in emergency care.

Working in the emergency unit makes people grow. I think everyone would have a coping mechanism [in dealing with WPV] at heart. After working here for long, we are bound to find a way to cope. The most important thing is to take good care of ED patients right! (N13)

I don’t want to blame patients. Maybe they just couldn’t control themselves at the time, for example, patients needing resuscitation, psychotic, drunk, these were the very reasons for them to come to the emergency unit. I now remind myself to watch out, and to be more cautious than before. The right thing is helping the patient quickly. (N4)

Emergency nurses identified peer support as a significant resource following an experience of WPV. Support from colleagues or managers, in the form of words or actions, helped them to recover and make a swift return to their work and lives. The following accounts are representative.

After encountering the [WPV] incident, I considered leaving the job. However, I received so many kind thoughts and words from colleagues and friends; sometimes just a note, a pat on the shoulder or a warm smile. It was really considerate of them. This is why I still love [working] here. (N13)

The HN [head nurse] immediately told me to go home and take some time off. She had my back all the time. I felt that was really what held me together and pulled me through. I then knew how to deal with this issue afterwards. (N16)

### Core Theme: Professional Commitment to Emergency Patient Care

**Figure 1:**
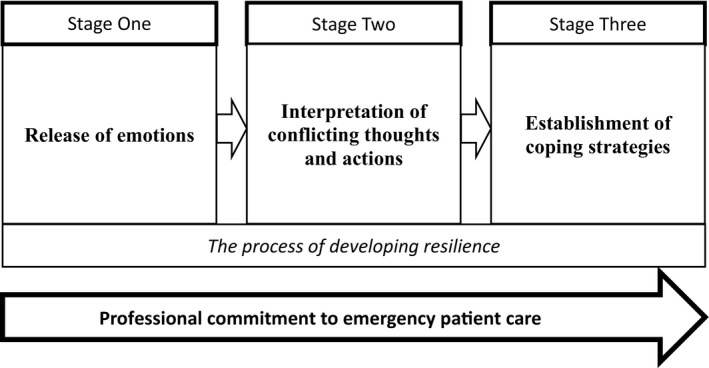
The process through which nurses develop resilience in relation to workplace violence in the emergency department.

In the early stage following an incident of WPV, ED nurses worked through their emotional responses to having experienced such an event while they were providing care to patients. Subsequently, they were able to strike a balance between positive and negative thoughts to support their ongoing commitment to nursing work. By drawing on support from colleagues and their own experience, they developed coping strategies to mitigate and, if necessary, manage future violent situations. Participants made frequent reference to their enthusiasm and dedication to emergency patient care. This professional commitment can be seen to underpin ED nurses’ motivation to provide quality emergency care to patients and emerged as the core theme in ED nurses’ accounts of their recovery from an encounter with WPV. This process of resilience is illustrated in Figure 1.

## Discussion

WPV has a range of psychological and emotional effects on ED nurses. Nurses who face WPV on a regular basis have been shown in previous research to accumulate increasingly negative emotions (Li et al., 2018). Exposure to WPV has been associated with a variety of deleterious psychological e?ects (Emergency Nurses Association, 2011; Magnavita, 2014), including frustration, fear, and anger (Hamdan & Abu, 2015). Such negative emotional responses can manifest as negative feelings towards nursing (Li et al., 2018). In the current study, ED nurses reported negative mood changes and other physio‐psychological responses to an encounter with WPV. These feelings could be projected outwardly to other patients in the ED and inwardly as nurses questioned its effect on their dedication to nursing.

Recent research on the resilience of ED nurses working in demanding units showed that, despite reporting negative feelings about the difficult situations they faced at work, doubt about the value of their profession, and a reduced passion for the work, the nurses remained optimistic about the job (Lin, Liang, Han, Chen, & Hsieh, 2019). ED nurses often need to weigh the pros and cons of their work, and make decisions to continue based on professionalism. In the present study, although participants reported conflicting thoughts, they were able to strike a balance between their negative feelings and their professional responsibilities.

Resilience is the ability to confront and adapt to stressful situations in ways that support a positive interaction with the environment. An individual who has developed resilience can learn from difficult situations and achieve both personal and professional growth as a result (Pangallo, Zibarras, Lewis, & Flaxman, 2015). While measuring the extent of resilience of ED nurses was outside the scope of the present study, nurses with greater adaptability toward a changing environment appeared better at recovering from setbacks in a highly stressful work environment such as the ED. The present study showed that ED nurses viewed professional growth as a foundation from which to recover from WPV. Participants developed their own coping strategies that enabled them to adapt to a stressful environment in a positive way following a WPV event. For example, during interviews participants mentioned that good communication skills and appropriate WPV management techniques were helpful for dealing with high‐risk patients. These characteristics are in keeping with the findings from the literature (Walsh, 2016) and suggestive of the need for future research on the role of resilience as a factor influencing how nurses respond to WPV.

Support from colleagues and supervisors was a valuable resource during the recovery process. This finding is consistent with those from other studies indicating that peer support and social networks are significant factors in the development of resilience (Hsieh, Chang, & Wang, 2017; Hsieh et al., 2016). Peer support is positively correlated with resilience, indicating that resilience is facilitated by the availability of a support system that has a variety of sources and dimensions (Sadler, Sarre, Tinker, Bhalla, & McKevitt, 2017; Sarre et al., 2014) and provides a positive environment. While previous studies show that nurses experiencing WPV have increased negative emotions over time (Li et al., 2018), the present study highlights the value of peer support from colleagues and supervisors and its role in mitigating at least some of the long‐term negative emotional effects of the WPV incidents.

Professional commitment has been defined as the extent to which an individual is dedicated to and proud of his or her profession, believes in its values and goals, and hopes to maintain membership thereof (Chang et al., 2015). Professional commitment provides a link between individuals and the professional values they have chosen to strive for (Ayaz‐Alkaya, Yaman‐Sözbir, & Bayrak‐Kahraman, 2018; Nesje, 2016). Commitment to nursing was an invisible motivator for ED nurses in the present study to continue to provide the best emergency care to patients. This is consistent with the principles of resilience theory in relation to the mechanisms that encourage personal growth, effective coping, and integration (Garcia‐Dia, DiNapoli, Garcia‐Ona, Jakubowski, & O’Flaherty, 2013). It has been suggested that nurses’ resilience can be enhanced through training and education that focuses on helping them to deal positively with the effects of WPV and minimize its negative impact on emergency patient care (Hollywood & Phillips, 2020).

## Limitations

The main limitation of the present study is that participants were recruited from three hospitals in Taiwan. The findings cannot necessarily be generalized to all emergency nurses. The inclusion criteria did not include anyone who left the ED due to WPV. This resulted in a more positive outcome. The present study did not seek to distinguish and draw comparisons between participants’ responses to physical assault and verbal assault in the ED. This may be a limitation of the study.

## Conclusions

WPV is frequently encountered in the ED, and immediately after such an event, nurses experience overwhelming negative emotions that affect their mental and physical well‐being. EDs should develop a strict but practical procedure to respond to WPV, and continue efforts to promote and enforce the policy of zero tolerance to violence. On the administrative level, hospitals should provide full institutional and executive support for nurses following an incident of WPV and acknowledge nurses’ professional dedication. Future studies should focus on WPV prevention, characteristics of nurse resilience, and the effect of WPV prevention and resilience training for nurses working in EDs. The present findings can inform the provision of practical support to nursing staff who have experienced WPV by alerting healthcare executives and institutions to their needs.

## Continuing Professional Development

*Journal of Nursing Scholarship* is pleased to offer readers the opportunity to earn Continuing Professional Development contact hours for select articles. This opportunity is valid for three years from each article's date of publication. Learn more here: https://www.sigmamarketplace.org/journaleducation
